# Identifying the Relationship Between Economic Prosperity and Quality of Life in Chronic Obstructive Pulmonary Disease Patients

**DOI:** 10.7759/cureus.40624

**Published:** 2023-06-19

**Authors:** Dimitris Mazetas, Mary Gouva, Athina Economou, Irini Gerogianni, Stefanos Mantzoukas, Konstantinos I Gourgoulianis

**Affiliations:** 1 Department of Respiratory Medicine, University General Hospital of Larissa, Larissa, GRC; 2 Faculty of Medicine, University of Thessaly, Volos, GRC; 3 Research Laboratory Psychology of Patients, Families & Health Professionals, University of Ioannina, Ioannina, GRC; 4 Department of Economics, University of Thessaly, Volos, GRC

**Keywords:** rehabilitation, birth order, economic, family, psychic health, physical health, intergenerational transmission of poverty, copd: chronic obstructive pulmonary disease, psychology, quality of life

## Abstract

Background

Chronic obstructive pulmonary disease (COPD) places a significant economic burden on national healthcare systems, and the economic effects of diseases have long been known. The study aimed to evaluate the association of parental family financial wealth with current economic prosperity and the combined effect of both on health-related quality of life (HRQOL) in a sample of patients with COPD. The moderating effect of birth order is further investigated.

Methods

The results of the study are based on a purposive sample of 105 COPD patients at the Larisa University Hospital pulmonology clinic (94 males and 11 females), with an average age of 68.9 (SD = 9.2). The data collection was carried out in the spring and summer of 2020. Participants completed the 36-item Short Form Survey (SF-36) and a sociodemographic questionnaire with self-reported parental and current wealth items. A mediation model with the moderation of the indirect effect of parental wealth on current wealth and the direct effect of parental wealth on HRQOL was applied to test the research hypotheses among the variables studied.

Results

Parental wealth was found to affect current wealth significantly, and both were involved considerably in HRQOL. Birth order had a significant moderating effect on the relationship between parental wealth and HRQOL. Among parental families with lower financial status, patients who grew up as third or later children had significantly lower HRQOL than the first or second children of these families. Neither age nor COPD duration was related to current wealth or HRQOL.

Conclusions

An intergenerational transmission of poverty was found in our sample. In addition, a birth order effect can provide further insight into the harsher environment that the later children of a low-income family are exposed to and the long-term implications for their HRQOL.

## Introduction

Chronic obstructive pulmonary disease (COPD) places a significant economic burden on national healthcare systems [1] and is the third leading cause of death worldwide, causing 3.23 million deaths in 2019 [2]. A recent cross-sectional study of adults over the age of 40 in a global sample of 41 urban and rural locations estimated the median prevalence of chronic airway obstruction to be 11.2% in males and 8.6% in females [3].

The economic differences between these diseases have long been known. For example, over 80% of COPD-related deaths occur in low- and middle-income countries without health systems that can adequately support patients [4]. Similar differences have also been reported in patients with COPD living in smaller geographic regions within countries with developed healthcare systems [5].

A primary goal of the literature on COPD is to elucidate the relationship between the overall quality of life of COPD patients and sociodemographic, biological, and physiological variables and symptoms of the disease. This research objective is of great importance given that poor quality of life is associated with a higher risk of hospitalization [6] and mortality [7].

Age [8], educational level [9], socioeconomic status and race [10], food insecurity [11], oxygen saturation, and shortness of breath [12] have been identified as factors affecting the well-being of COPD patients. Gender is not equally proven as a discriminating factor, as reports show significant and insignificant differences in quality of life between men and women with COPD [9,13].

Research has shed light on various factors related to patients' quality of life, but there is still much to be said. The present study aims to provide a new perspective by proposing a model that links these patients' health-related quality of life (HRQOL) to their parental family's financial wealth and current financial situation while considering their age and disease duration. In this context, and because of several studies showing that wealth varies with parental wealth and birth order [14,15], in the current study, birth order is also considered as a possible moderator of the relationship between parental wealth and current wealth and between parental wealth and HRQOL.

## Materials and methods

Hypothesis development

In the context of COPD, it has recently been reported that people living below the relative poverty line have an increased prevalence of COPD, while older people living below the poverty line are at greater risk of developing COPD than those living above the poverty line [16]. In addition, this gap appears to be widening, because although age- and sex-standardized all-cause mortality in COPD patients fell by 35% from 1996 to 2012, the positive change was more significant in people with the highest socioeconomic status versus those with the lowest socioeconomic status, resulting in increasing inequality between rich and poor [17].

Against this background, we aimed to investigate the direct effect of financial security with HRQOL in patients with COPD in this study. Therefore, our first goal is to prove the following research hypothesis:

 H1: Current wealth will be significantly related to HRQOL.

Parental family wealth relative to current wealth and the long-term effects of parental family wealth on HRQOL has not yet been investigated in this population group. Our second goal is, therefore, to prove the following research hypotheses:

H2: There is no relation between parental wealth and the current wealth of the patient.

H3: There is no significant direct impact of parental wealth on the overall HRQOL.

Barclay and Kolk [18] have raised important questions about the impact of birth order on the effects of paternal wealth on current wealth and the long-term implications of paternal wealth on the felt experience of quality of life in adulthood.

Accordingly, for this study, it was of interest to investigate the following research hypotheses:

H4a: There will be a significant differentiation in current wealth according to the patient's birth order.

H4b: Birth order moderates the relationship between parental wealth and current wealth, with a more significant impact of parental wealth on the current wealth of firstborn children.

H5a: There is significant differentiation in HRQOL according to the patient's birth order.

H5b: Birth order moderates the relationship between parental wealth and HRQOL, with firstborn children having a greater long-term impact on HRQOL than later-born siblings.

Aging individuals have greater physical, cognitive, and mental health deterioration. Nonetheless, there is ample evidence in the relevant literature that older adults outperform younger adults in making shared financial decisions because of their experiential knowledge and reduced negative emotional responsiveness over time [19]. It is, therefore, reasonable to assume that this economic wisdom is reflected in the significant effect of age on current wealth. In addition, suffering from a chronic illness is expected to affect work productivity, which in turn affects the personal financial situation. The present study aims to show that age and the duration of illness do not have as significant an impact on current assets as the financial starting position determined by the parent's assets. In particular, we want to show that:

H6a: The effect of age on current wealth is not as significant as the corresponding effect of parental wealth.

H7a: The effect of illness duration on current wealth is not as significant as the corresponding effect of parental wealth.

An analogous decline in mental health accompanies the inevitable decline in physical and cognitive function. Therefore, it is reasonable to assume that suffering from a chronic disease such as COPD over a more extended period would worsen the self-assessed quality of life. In contrast, increasing age is associated with improvements in various mental health attributes. This has been particularly confirmed in COPD patients, where younger patients have been found to have a poorer health-related quality of life than older individuals [20]. Therefore, one aim of the present study is to evaluate the extent to which financial wealth outweighs age and disease duration concerning their influence on HRQOL. To this end, we tested the following research hypothesis:

H6b: The effect of age on HRQOL is not as significant as the corresponding effect of current wealth.

H7b: The effect of illness duration on HRQOL is not as significant as the corresponding effect of current wealth.

Theoretical model

Overall, our model assesses the intergenerational transmission of the poverty hypothesis and the impact of birth order on a sample of COPD patients. In particular, we aim to confirm that a lack of resources in the parental family reduces parents' ability to adequately care for late-born children, with consequences that affect their long-term health. In addition, we assume that birth order moderates the parental wealth effect in favor of the first two children.

**Figure 1:**
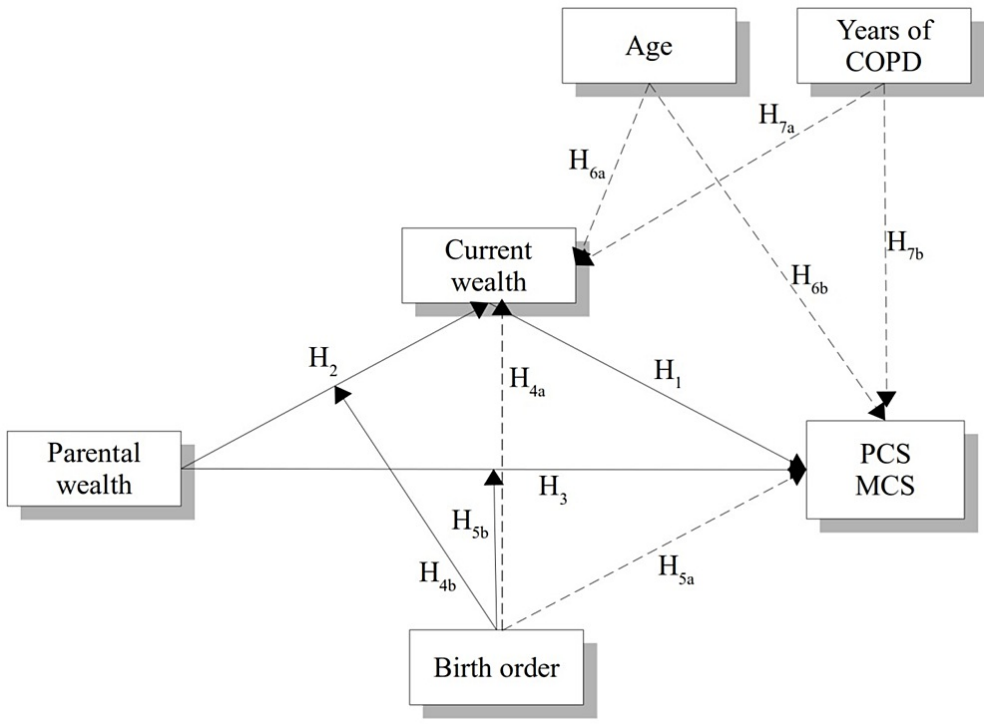
The conceptual model. PCS: physical component score, MCS: mental component score, COPD: chronic obstructive pulmonary disease.

In summary, we test the mediation model of Figure 1, in which current wealth mediates the relationship between parental wealth and adult HRQOL and birth order mediates the relationship between parental and current wealth, and the relationship between parental wealth and HRQOL, also controlling for age and duration of COPD disease.

Participants and study design

The research team selected the participating patients through purposive sampling at the pulmonary clinic of Larisa University Hospital in Greece. Any adult COPD patient who attended the hospital for regular COPD check-ups was eligible to participate in the study. Before completing the questionnaire, the patients were informed about the purpose of the study and gave their consent. Each participant completed a sociodemographic questionnaire, which included a pair of questions to assess the parental and current economic situation on a four-point Likert scale. In addition, participants were asked to complete the 36-item Short Form Survey (SF-36), which was used to self-assess their general health.

**Figure 2:**
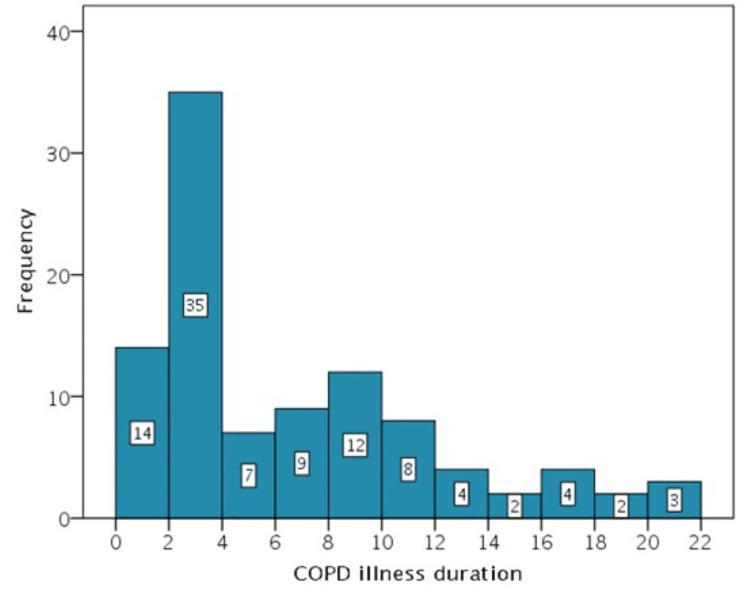
COPD disease duration. COPD: chronic obstructive pulmonary disease.

The data collection was carried out in the spring and summer of 2020. One hundred thirty-nine Greek adults took part in the study. Of these, 34 were excluded from the evaluation due to incomplete information in the sociodemographic questionnaire (Figure 2).

Most of them were married (N = 83, 89%), pensioners (N = 81, 77.1%), graduated from primary school (N = 63, 60%), and lived in Thessaly (N = 57, 54.3%). Sixty-two (59%) reported walking daily, and 50 (47.6%) spent time caring for their garden. Further, 34 (32.4%) said they could financially afford summer vacations. The birth order in their paternal family ranged from one to eight (Table 1), and the self-evaluated parental and current wealth of their families is presented in Table 2. 

**Table 1:** Birth order of the participants (N, %).

First	Second	Third	Fourth	Fifth	Sixth	Seventh	Eighth
40 (38.1%)	17 (16.2%)	27 (25.7%)	12 (11.4%)	4 (3.8%)	2 (1.9%)	1 (1.0%)	2 (1.9%)

**Table 2:** Parental wealth and current wealth of the participants.

Wealth	1: Lower	2: Middle	3: Upper	4: High	5: Highest	Total
Parental wealth	36 (34.3%)	36 (34.3%)	30 (28.6%)	3 (2.9%)	0 (0%)	105 (100%)
Current wealth	11 (10.5%)	35 (33.3%)	43 (41%)	15 (14.3%)	1 (1%)	105 (100%)

Measurements

Participants completed a sociodemographic questionnaire that included self-assessment items about leisure activities (walking, gardening, and summer vacations), parental wealth, and their current wealth. In addition, the 36-item Short Form Survey (SF-36) was completed, which was used to assess their health-related quality of life.

The SF-36 Health Survey [21] is a short-form, multipurpose, 36-item health survey. The general health of the respondent is assessed using eight scales (P.F.: physical functioning, R.P.: role limitations due to physical health, B.P.: physical pain, G.H.: general health perceptions, RE: role limitations due to emotional health, VT: vitality, MH: mental health, S.F.: social role function). Two composite scores representing overall physical (PCS) and mental (MCS) health are calculated from the eight scales by summing P.F., R.P., B.P., G.H., RE, VT, MH, and S.F., respectively.

Data re-coding

The low frequency of some responses related to birth order, parental wealth, and current wealth required re-coding these variables prior to statistical analysis. Therefore, for the purposes of the study, birth order was converted into a binary variable with values ​​0 (first and second birth order; N = 57; 54.3%) and 1 (third or higher birth order; N = 48; 45.7%). In addition, categories 3 (N = 30) and 4 (N = 3) of parental wealth were merged into a single category (upper) with a frequency of 33 (31.4%), resulting in a three-valued homogeneous parental wealth variable (c2(2) = 0.171, p = 0.918). Finally, categories 4 (N = 15) and 5 (N = 1) of current assets were merged into a single category (high) with a frequency of 16 (15.2%).

Ethics

The University of Larisa, Department of Medicine Ethics Committee (12/2/2019) approved the study. The study was conducted following the Helsinki Declaration for ethical principles for medical research involving human subjects. All participants were informed of the purpose of the investigation, assured of the confidentiality of all personal data, and gave their written consent. The questionnaires were completed by the participants in the researcher's presence and with his help when necessary.

Statistical analysis

To test the theoretical model, a mediation model was applied to moderate the indirect effect of parental wealth on current wealth and the direct effect of parental wealth on HRQOL (Figure 1). Model 8 of the PROCESS function for R [22] was applied twice, using the physical (PCS) and mental (MCS) components of the SF-36 scales as the dependent variables. Parental wealth was positioned as the independent variable, current wealth was the mediator, and age and disease duration were defined as covariates of current wealth and as the model's dependent variable (PCS or MCS).

Birth order has been positioned as a moderator of the relationship between parental wealth and current wealth and between parental wealth and PCS and MCS, respectively. In both applications of the model, the parental wealth variable was replaced by two dummy variables aimed at comparing the middle and upper financial categories to the lower one. The statistical significance of the indirect effects of parental wealth on HRQOL (via current health status) was assessed using 5,000 bootstrap samples to generate bias-adjusted 95% confidence intervals.

The analysis assessed the effect of the current financial situation on HRQOL. The moderating effect of birth order on the relationship between parental wealth and current wealth. The indirect effects of parental wealth on HRQOL via current wealth. The moderating effect of birth order on the relation between parental wealth and HRQOL. All data were analyzed using the SPSS statistical package (version 21) [23] and the R statistical language equipped with the PROCESS function [22].

## Results

The results of the present study were obtained from a sample of 105 patients, 94 men (89.5%) and 11 women (10.5%), aged 42 to 87 years (M = 68.9, SD = 9.2). They were diagnosed with COPD one to 21 years before study participation (Figure 2, mode = two years, median = four years, mean = 6.1 years, SD = 5.3 years). The two summary component scores (PCS and MCS) were significantly positively correlated (Pearson's r(105) = 0.894, p < 0.001). As suggested in the relevant literature [24], PCS and MCS were confirmed to adequately represent the eight subscales of the SF-36 questionnaire (Table 3). A single exception was the vitality subscale, which was not significantly related to the scores of either component, a finding not reported for the first time and questions on the validity of the specific subscale raises in samples from patients suffering from a chronic disease.

**Table 3:** Pearson’s correlation between component summaries and subscales of the SF-36. SF-36: Short Form (36) Health Survey; M: mean; SD: standard deviation; PCS: physical component summary scores; MCS: mental component summary scores; PF: physical functioning; RP: physical role; BP: bodily pain; GH: general health; RE: emotional role; VT: vitality; MH: mental health; SF: social role; **: correlation is significant at the 0.01 level (two-tailed).

Component summary scores	Sections of SF-36	PF	RP	BP	GH	RE	VT	MH	SF
	M (SD)	57.6 (29.4)	68.1 (45.3)	84.9 (27.7)	53 (25.6)	69.2 (46.2)	57.6 (13.3)	66.1 (21.7)	72.1 (38.2)
PCS	263.5 (103.1)	0.837^**^	0.872^**^	0.729^**^	0.736^**^	0.839^**^	−0.110	0.577^**^	0.841^**^
MCS	265.1 (91.5)	0.745^**^	0.910^**^	0.534^**^	0.560^**^	0.914^**^	0.026	0.615^**^	0.935^**^

Wealth, HRQOL, and leisure activities

The quality of life summarizing scores (PCS and MCS) were significantly positively correlated to current wealth (PCS: r(105) = 0.277, p < 0.01, MCS: r(105) = 0.257, p < 0.01). The 62 patients that responded to walking regularly as a physical exercise were found to have a significantly higher PCS than the rest 43 (285.7 ± 96.0 vs 231.6 ± 105.8, t(103) = 2.729, p = 0.007), while the 34 patients that could financially afford summer vacations had a significantly higher MCS than the 71 respondents that couldn't afford so (293.0 ± 83.0 vs 251.7 ± 93.0, t(103) = 2.204, p = 0.030).

Evaluation of the effects on current wealth in the context of the tested model

**Figure 3:**
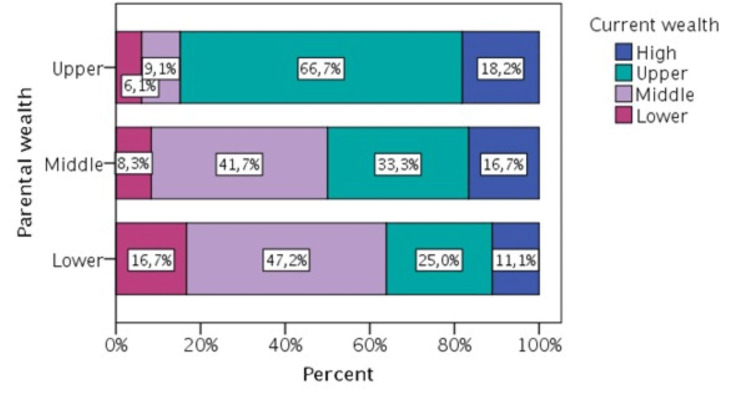
Concurrences of parental and current wealth self-evaluation.

A significant positive effect of parental wealth on current wealth was found among participants raised in the wealthiest families compared to those with the lowest income families (upper level: b = 0.695, p = 0.013, 95% CI 0.148-1.243). Thus, the research hypothesis H2 is partially supported (Figure 3, c2(6) = 19.454, p = 0.003).

On the other hand, no significant effect was reported for birth order (p = 0.749), implying that research hypothesis H4a is not supported. Furthermore, age (p = 0.055) and disease duration (p = 0.394) were also not significant explanatory variables, so the research hypotheses H6a and H7a are validated (Table 4). Furthermore, no significant interaction was found between birth order (BRD) and parental wealth (WLTP) at current health (middle level × birth order: p = 0.467 and upper level × birth order: p = 0.851), implying that research hypothesis H4b is not supported. 

**Table 4:** Prediction model of current wealth (1). B: beta coefficients from linear models; SE: standard error; t: student's t-test; p: p-value; CI: confidence interval; (1): R2 = 0.151, F(7, 97) = 2.456, p = 0.023; (2): reference level: lowest parental wealth category.

Effect	B	S.E.	T	p	95% CI	
					Lower	Upper
Constant	0.958	0.679	1.410	0.162	−0.390	2.306
Parental wealth						
Middle^(2)^	0.432	0.277	1.561	0.122	−0.117	0.982
Upper^(2)^	0.695	0.276	2.520	0.013	0.148	1.243
Birth order	−0.091	0.282	−0.321	0.749	−0.651	0.470
Parental wealth × birth order						
Middle^(2)^ × birth order	−0.287	0.394	−0.730	0.467	−1.068	0.494
Upper^(2)^ × birth order	−0.078	0.412	−0.188	0.851	−0.896	0.741
Age	0.019	0.010	1.942	0.055	−0.000	0.038
Illness duration	0.014	0.016	0.856	0.394	−0.019	0.047

Evaluation of the effects on HRQOL in the context of the tested model

**Figure 4:**
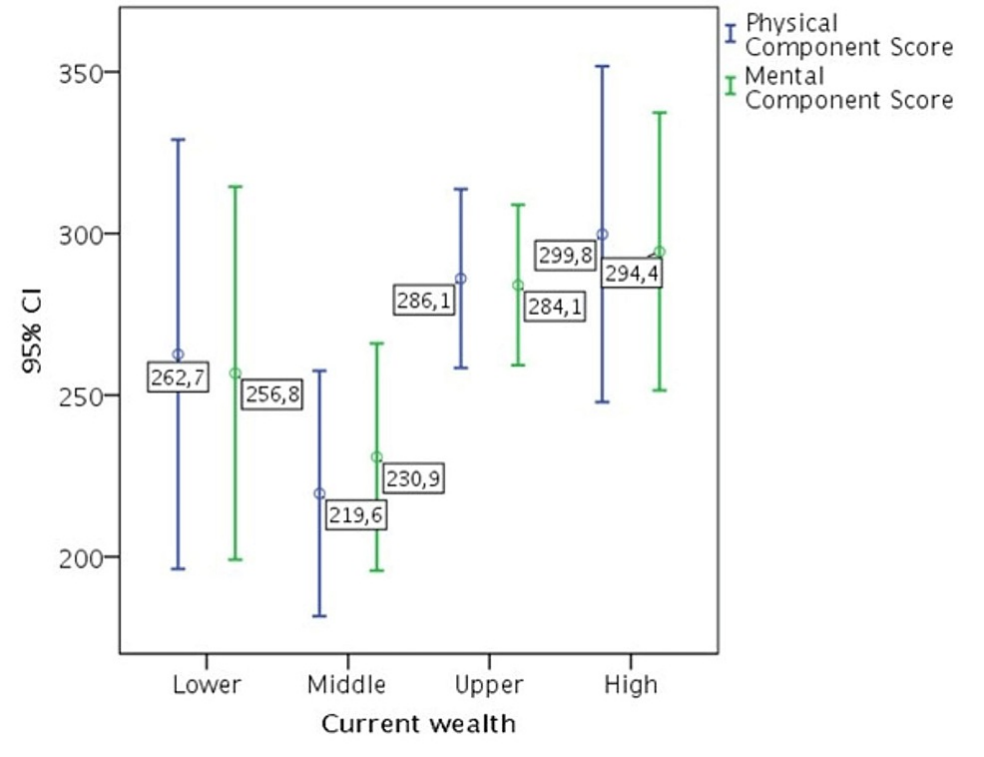
Current wealth effect on health-related quality of life.

Current wealth was found to have a significant effect on both physical (b = 26.888, p = 0.027, 95% CI 3.130-50.646) and mental health (b = 22.638, p = 0.033, 95% CI 1.914-43.363), supporting research hypothesis H1 (Table 5). In particular, patients who belonged to the upper and high wealth categories were characterized by higher scores on both health component scales than those in the lower and intermediate wealth categories (Figure 4). 

**Table 5:** Prediction models of physical and mental component summaries. B: beta coefficients from linear models; t: student's t-test; p: p-value; CI: confidence interval; (1): PCS: R2 = 0.166, F(8, 96) = 2.384, p = 0.022; MCS: R2 = 0.194, F(8, 96) = 2.897, p = 0.006; (2): reference level: lowest parental wealth category.

Effect		Physical component summary (PCS)^(1)^					Mental component summary (MCS)^(1)^				
		B	t	p	95% CI		B	t	p	95% CI	
					Lower	Upper				Lower	Upper
Constant		258.847	3.200	0.002	98.300	419.393	248.460	3.522	0.001	108.414	388.506
Parental wealth											
Middle^(2)^	Birth order 1-2	−26.824	−0.812	0.419	−92.434	38.785	−11.142	−0.386	0.700	−68.374	46.090
	Birth order ≥3	72.748	2.163	0.033	5.985	139.511	94.995	3.238	0.002	36.757	153.233
Upper^(2)^	Birth order 1-2	−40.168	−1.197	0.234	−106.799	26.463	−32.317	−1.104	0.272	−90.440	25.806
	Birth order ≥3	102.548	2.742	0.007	28.324	176.773	90.331	2.769	0.007	25.584	155.077
Indirect via current wealth											
Current wealth		26.888	2.246	0.027	3.130	50.646	22.638	2.168	0.033	1.914	43.363
Birth order		−88.469	−2.657	0.009	−154.552	−22.387	−90.406	−3.113	0.002	−148.051	−32.762
Parental wealth × birth order											
Middle^(2)^ × birth order		99.572	2.140	0.035	7.220	191.924	106.137	2.615	0.010	25.577	186.696
Upper^(2)^ × birth order		142.716	2.935	0.004	46.182	239.251	122.648	2.891	0.005	38.440	206.856
Age		−0.251	−0.216	0.830	−2.562	2.059	−0.061	−0.060	0.953	−2.076	1.955
Illness duration		−3.119	−1.606	0.111	−6.972	0.735	−2.388	−1.410	0.162	−5.750	0.974

Age and disease duration were not significantly related to the physical (PCS) and mental (MCS) components of HRQOL; thus, research hypotheses H6b and H7b are also validated.

Birth order as a moderator between parental wealth and HRQOL

Patients in both middle and upper parental wealth groups who were born in the third or higher position benefited significantly more from parental wealth for their physical (PCS: pMiddle, BRD ≥ 3 = 0.033, pUpper, BRD ≥ 3 = 0.007) and mental health (MCS: pMiddle, BRD ≥ 3 = 0.002, pUpper, BRD ≥ 3 = 0.007) than the patients in the lower parental wealth group (Table 5).

**Figure 5:**
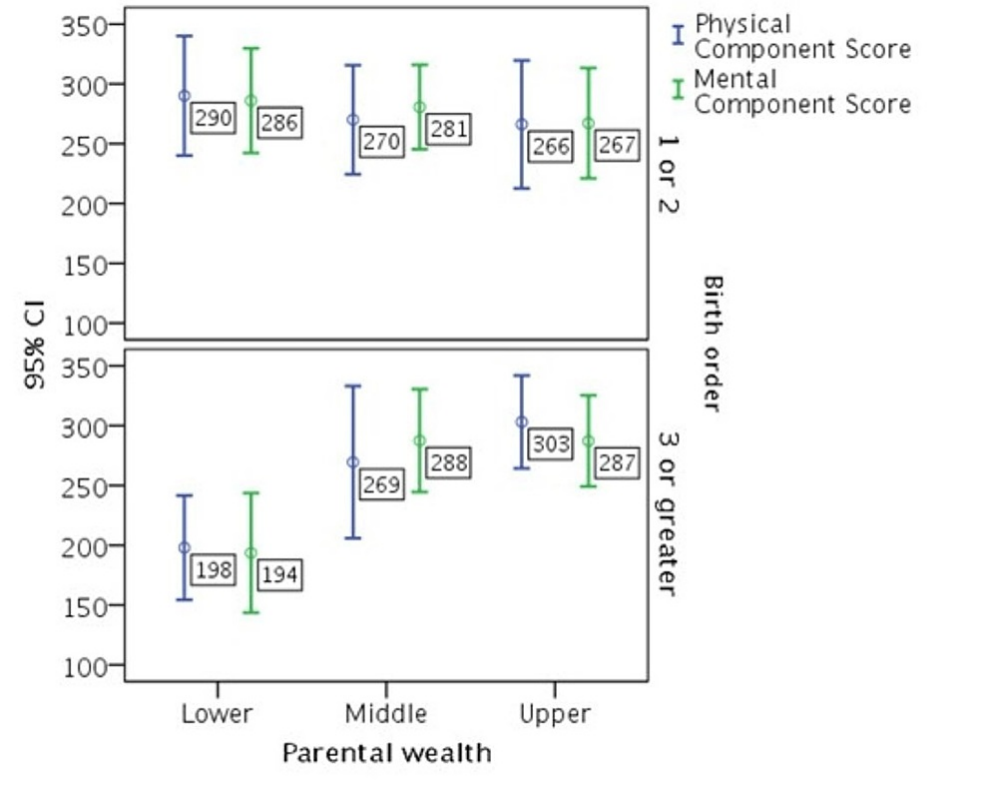
Interaction of birth order and parental wealth on health-related quality of life.

In contrast, no significant differences were reported for patients born as first or second children among the three wealth categories (PCS: pMiddle, BRD < 3 = 0.419, pUpper, BRD < 3 = 0.234 and MCS: pMiddle, BRD < 3 = 0.700, pUpper, BRD < 3 = 0.272) (Table 5). In particular, research hypothesis H5b, suggesting a moderating effect of birth order on parental wealth and HRQOL relationship, is supported, while both hypotheses H5a and H3 suggest unconditioned effects of birth order and parental wealth on HRQOL, which are not validated. The birth order moderation effect is portrayed in Figure 5.

Indirect parental wealth effect on HRQOL

Bootstrap analysis (Table 6) showed a significant indirect parental wealth effect on HRQOL for patients grown up in the upper parental wealth group. This positive effect was found to be significant for the patients having been born as first or second children (bPCS = 18.689, 95% CI 0.416-42.460, bMCS = 15.735, 95% CI 0.538-34.302), although there was a minor difference with the patients being third or higher-positioned children in the upper parental wealth group. That is, an unconditional long-term parental wealth effect on HRQOL for the upper parental wealth group is suggested, an effect that is also necessary to be confirmed in a later study.

**Table 6:** Indirect effects of parental wealth on health-related quality of life via current wealth. B: beta coefficients from linear models; CI: confidence interval; (1): reference level: lowest parental wealth category.

Parental wealth category	Physical component summary (PCS)			Mental component summary (MCS)		
	B	95% CI		B	95% CI	
		Lower	Upper		Lower	Upper
Middle^(1)^						
Birth order 1-2	11.62	−5.29	34.16	9.78	−5.11	28.32
Birth order ≥3	3.90	−12.26	20.93	3.28	−10.33	16.97
Difference	−7.72	−36.37	14.96	−6.50	−29.55	13.70
Upper^(1)^						
Birth order 1-2	18.69	0.42	42.76	15.74	0.54	34.30
Birth order ≥3	16.60	−0.06	40.40	13.98	−0.20	33.17
Difference	−2.09	−24.52	23.07	−1.76	−20.25	19.77

Bootstrap analysis (Table 6) showed a significant indirect influence of parental wealth on HRQOL for patients in the upper parental wealth group. This positive effect proved to be significant for the patients born as a first or second child (bPCS = 18.689, 95% CI 0.416-42.460, bMCS = 15.735, 95% CI 0.538-34.302). However, there was a small difference among those patients who were the third or higher children in the parents' upper wealth group. That is, an unconditional long-term effect of parental wealth on HRQOL is proposed for the upper parental wealth group, an effect that also needs to be confirmed in a later study.

The findings of the study are summarized in Table 7.

**Table 7:** Evidence providing from the data for the research hypotheses of the study.

Research hypothesis		Supported by the data
H_1_	Current wealth is significantly related with HRQOL.	Yes
H_2_	Parental wealth is positively related to a patient's current wealth.	Partially
H_3_	There is a significant direct long-term effect of parental wealth on HRQOL.	No
H_4a_	There is significant differentiation in current wealth accordingly to birth order.	No
H_4b_	Birth order moderates the relation between parental wealth and current wealth, with a greater effect of parental wealth to current wealth for the firstborn children.	No
H_5a_	There is significant differentiation in HRQOL accordingly to birth order.	No
H_5b_	Birth order moderates the relation between parental wealth and HRQOL, with first-born children to have a greater long-term effect on HRQOL than the later-born siblings.	Yes
H_6a_	The effect of age on current wealth is not as significant as the corresponding parental wealth effect.	Yes
H_7a_	The effect of illness duration on current wealth is not as significant as the corresponding parental wealth effect.	Yes
H_6b_	The effect of age on HRQOL is not as significant as the corresponding current wealth effect.	Yes
H_7b_	The effect of illness duration on HRQOL is not as significant as the corresponding current wealth effect.	Yes

## Discussion

The literature has sufficiently demonstrated that the economically weakest patients suffer most from COPD [25]. Greece, Bulgaria, Romania, and Spain comprise a group of European countries where over one-quarter of the population is estimated to be at risk of poverty or social exclusion due to financial insecurity [26]. Specifically, in Greece, the percentage of citizens living in poverty has been reported to increase during the recent and ongoing economic crisis [27], setting an apparent harsher environment for the patients to effectively manage the illness [4]. In that context, the disadvantaged position of the economically weaker was reflected in the sample of the present survey, where the vast majority of the respondents identified their financial situation as poor. Further, the finding that economic prosperity is positively related to health-related quality of life (HRQOL) confirms previous results among COPD patients, manifesting the implications of poverty for another time.

Financial well-being enhances patients' ability to address their physical, social, and emotional aspects, maintaining better physical and mental health [28]. In the context of the present study, the financial ability to do summer vacations was positively related to the mental health component. This finding further demonstrates the positive impact of financial health on the overall quality of life, even in the presence of chronic disease. In particular, it is indicated that financial health enhances the ability of a COPD patient to maintain social interactions and a sense of normality, freedom, and purpose, which is reported to affect the overall quality of life in the context of COPD disease [29].

Illness duration of a chronic illness is reported to worsen professional autonomy and decrease patient's chances of attaining a normal professional life, worsening their perceived quality of life [9]. Furthermore, specifically among COPD patients, the patient's age is reported to have a negative impact on quality of life [9]. Interestingly, in the sample of the present study, which consisted mainly of pensioners of lower socioeconomic level, both illness duration and age effects were not confirmed, indicating interaction effects between age, professional status, and illness duration on quality of life, a research goal that deserves to be studied further in future research.

A novel contribution of the present study is highlighting the direct and indirect effects of parental wealth on HRQOL. That is, the intergenerational transmission of socioeconomic disadvantage that has long been reported in the literature is yet again confirmed [30]. In particular, it is suggested that the parental family's wealth provides the offspring with a significant long-term advantage over individuals who grow up in poorer families in terms of the capability to maintain an adequate quality of life during their later years, even in the event of a chronic illness such as COPD. This relationship should not be realized as a direct cause-and-effect phenomenon, as numerous life decisions and events between the early and later years of a person's life affect the final health-related quality of life. However, it is adequately manifested that a person's evolution into life strongly depends on the economic environment in which they grew up. In that context, age and illness duration had insignificant effects on current wealth and HRQOL, showing that parental wealth was a variable of greater importance.

Among financially strained individuals, differences in health behaviors, sociopolitical factors, and social and structural environmental exposures have been sufficiently shown to be related to the illness's evolution [30]. Towards the further enlightenment of this relation, an additional contribution of the present study is the indication of birth order as a factor associated with long-term quality of life among COPD patients. In particular, it is suggested that the supporting ability of a low-income family in terms of material and financial support is reduced significantly for later children, forcing the parents to compromise and provide the later children with the available resources rather than the desired ones. Specifically, it is confirmed that among the children of low-income families, the younger ones are disadvantaged compared to the first children of the family, with long-term consequences [18].

The prevalence and long-term consequences of COPD have long been shown to depend significantly on the state's economic well-being [4]. Overall, the results of this study confirm the disparity between the rich and poor concerning the benefit of positive scientific developments in the treatment of COPD [17].

Strengths and limitations

The present study provides a new perspective by proposing a model that links these patients' health-related quality of life to their parental family's financial wealth and current financial situation. Also, birth order was shown as a possible moderator of the relationship between parental wealth and current wealth and between parental wealth and quality of life.

There are some limitations of the study that should be reported. First, parental and current wealth was subjectively self-assessed using a single response from each participant on a five-point Likert scale, a relatively simple way of describing an individual's overall financial situation. A composite index containing various measures of economic well-being would more accurately reflect financial health. However, no reliable historical data on patients' parental wealth could be reported during the sampling process. Therefore, using Likert scale responses to measure financial well-being was decided. Quality of life was also self-evaluated using SF-36, so questions are raised about the report's credibility, although it is generally considered a valid tool. Finally, the sample size for this type of analysis was relatively small, indicating that the analytical results were less meaningful. Repeating the study in a larger sample will clarify the results of this study with greater accuracy.

## Conclusions

This study provides additional information on the role of wealth in managing COPD disease from the patient's perspective. The influence of the parental family's financial wealth on the long-term quality of life of the offspring is demonstrated, an effect that is not related to the age and duration of the disease.

Finally, it was found that the younger children in large, low-income families are disadvantaged in the case of a chronic condition such as COPD, a finding suggesting that financial difficulties may be related to inadequate parental care for later children. Our findings could assist in the development of political and social actions aimed at the financial and social assistance of chronic sufferers.
